# Biodegradable Polyhydroxyalkanoates with a Different Set of Valerate Monomers: Chemical Structure and Physicochemical Properties

**DOI:** 10.3390/ijms241814082

**Published:** 2023-09-14

**Authors:** Tatiana G. Volova, Natalia O. Zhila, Evgeniy G. Kiselev, Aleksey G. Sukovatyi, Anna V. Lukyanenko, Ekaterina I. Shishatskaya

**Affiliations:** 1 Institute of Biophysics SB RAS, Federal Research Center “Krasnoyarsk Science Center SB RAS”, 50/50 Akademgorodok, Krasnoyarsk 660036, Russia; volova45@mail.ru (T.G.V.); evgeniygek@gmail.com (E.G.K.); a.sukovatiy@yandex.ru (A.G.S.); shishatskaya@inbox.ru (E.I.S.); 2 Basic Department of Biotechnology, School of Fundamental Biology and Biotechnology, Siberian Federal University, 79 Svobodnyi Av., Krasnoyarsk 660041, Russia; lav@iph.krasn.ru; 3 L.V. Kirensky Institute of Physics SB RAS, Federal Research Center “Krasnoyarsk Science Center SB RAS”, 50/38 Akademgorodok, Krasnoyarsk 660036, Russia

**Keywords:** copolymers, P(3HB-*co*-3HV-*co*-4HV), P(3HB-*co*-3HV-*co*-3H4MV), physicochemical properties, thermal behavior, isothermal crystallization, spherulites formation rate, morphology

## Abstract

The properties, features of thermal behavior and crystallization of copolymers containing various types of valerate monomers were studied depending on the set and ratio of monomers. We synthesized and studied the properties of three-component copolymers containing unusual monomers 4-hydroxyvalerate (4HV) and 3-hydroxy-4-methylvalerate (3H4MV), in addition to the usual 3-hydroxybutyrate (3HB) and 3-hydroxyvalerate (3HV) monomers. The results showed that P(3HB-*co*-3HV-*co*-4HV) and P(3HB-*co*-3HV-*co*-3H4MV) terpolymers tended to increase thermal stability, especially for methylated samples, including an increase in the gap between melting point (T_melt_) and thermal degradation temperature (T_degr_), an increase in the melting point and glass transition temperature, as well as a lower degree of crystallinity (40–46%) compared with P(3HB-*co*-3HV) (58–66%). The copolymer crystallization kinetics depended on the set and ratio of monomers. For terpolymers during exothermic crystallization, higher rates of spherulite formation (G_max_) were registered, reaching, depending on the ratio of monomers, 1.6–2.0 µm/min, which was several times higher than the G_max_ index (0.52 µm/min) for the P(3HB-*co*-3HV) copolymer. The revealed differences in the thermal properties and crystallization kinetics of terpolymers indicate that they are promising polymers for processing into high quality products from melts.

## 1. Introduction

The widespread use of synthetic plastics is accompanied by an increase in the amount of plastic waste. This has become a global environmental problem [1,2,3], which requires an immediate solution [4,5]. The solution to the problem of plastic waste is associated today with the need to recycle it, despite the large labor and financial costs required for this [6]. It is believed that the organization and implementation on a global scale of a closed plastic cycle will significantly reduce plastic pollution of the biosphere, and will also help reduce the greenhouse footprint and greenhouse gas emissions [7,8].

The second way to solve the problem of plastic waste is a gradual transition to destructible polymeric materials of a new generation [9,10,11]. A special place among degradable polymers of synthetic and natural origin is occupied by polymers of microbiological origin, called polyhydroxyalkanoates (PHAs). PHAs are a family of biodegradable thermoplastic polymers of various chemical structures with different physicochemical properties [12,13,14,15,16,17].

Polyhydroxyalkanoates have a high potential for various applications, from reconstructive medicine to communal and agriculture [18,19,20]. Microorganisms are capable of synthesizing these polymers using various substrates including industrial, domestic, and agricultural waste. Therefore, PHAs have great potential to contribute to “The Circular Economy” [21,22,23].

Poly(3-hydroxybutyrate) [(P(3HB)] was the first polyhydroxyalkanoate to be discovered. Inclusions of P(3HB) in the form of granules in bacteria were observed by Beijenrinck in 1888 [24]. The first study of this polymer was carried out much later [25]. The author of this work isolated two components from the bacteria *Bacillus megaterium*, which, in his opinion, were the product of poly(3-hydroxybutyrate) hydrolysis. Both products had the empirical formula (C_2_H_3_O_3_), one of them crystallized, had a melting point at 120 °C, the other was amorphous and melted at 157 °C. Lemoingne later proved that both components are poly(3-hydroxybutyrate), but have different molecular weights. Pure P(3HB), however, has a high crystallinity of 70% and higher, it is brittle and poorly resistant to stretching. Insufficient elasticity and thermal stability of P(3HB) complicate the processes of its processing, which limits the possible areas of application [26,27].

If poly(-3-hydroxybutyrate) was the only polyhydroxyalkanoate, it most likely would not have great prospects. However, a polymer was isolated from activated sludge, the properties of which differed from those of P(3HB). Chromatographic analysis showed, in addition to the dominant monomers of 3-hydroxybutyric acid, the presence of monomers of 3-hydroxyvaleric, 3-hydroxyhexanoic, and, possibly, 3-hydroxyoctanoic acids as minor components [28]. It was the first heteropolymer member of the polyhydroxyalkanoate family. It was found that the presence of 3-hydroxyvalerate monomers in PHA significantly affects the characteristics, reducing the melting point and crystallinity of the material, making it more elastic and convenient for processing compared with P(3HB) [29].

The discovery of the ability of microorganisms to synthesize PHA heteropolymers has become a strong impetus for expanding research on these biopolymers. Quite quickly, it was shown that PHAs of various chemical compositions have different structures and basic physicochemical properties, including molecular weight, degree of crystallinity, mechanical strength, biodegradation rates, etc. [30,31,32,33,34,35]. This opens up prospects for the targeted synthesis of biopolymers with desired properties. To date, more than 150 types of various polyhydroxyalkanoates are known, among which only a small number of PHAs have been well studied [36]. Professor Alexander Steinbüchel proposed to divide the variety of known PHAs into three groups based on the length of the carbon chain—short-chain length (SCL), consisting of acids with a carbon chain length of three to five carbon atoms; medium-chain length (MCL), which contain from 6 to 14 carbon atoms, and long-chain length (LCL), containing acids C17 and C18 [37]. Among the actively studied PHA copolymers are two-component polymers containing, in addition to 3-hydroxybutyrate (3HB) monomers, monomers of 4-hydroxybutyrate (4HB), 3-hydroxyvalerate (3HV) or 3-hydroxyhexanoate (3HHx), as well as three- and four-component PHA containing these monomers in various ratios.

However, most actively studied bacterial polyhydroxyalkanoates, including poly(3-hydroxybutyrate) P(3HB), copolymers of poly(3-hydroxybutyrate-*co*-3-hydroxyvalerate) P(3HB-*co*-3HV) and poly(3-hydroxybutyrate-*co*-3-hydroxyhexnoate) P(3HB-*co*-3HHx) are characterized by slow crystallization and require a long solidification time after melting during molding [38]. It complicates the processing of these polymers from melts and adversely affects the quality of the resulting products. Therefore, the search and study of other PHA representatives is relevant in order to identify more technological types of polymers characterized by higher crystallization rates and providing higher quality products. For example, a copolymer of (3-hydroxy-2-methylbutyrate) P(3H2MB) has been described that exhibits rapid crystallization and the highest thermal stability among the known types of PHA; its melting point is 197 °C [39,40]. Another new type of PHA synthesized using tigloic acid has a higher affinity for crystallization than the previously described scl-PHA [41].

As defined by the authors of [42], these new types of PHA are the so-called unusual PHA. The authors suggested that unusual PHAs include a wide range of little-studied types of PHAs. Among them are PHAs of microbial origin, which are synthesized either from natural monomers bearing various functional groups, or from their chemical derivatives, as well as polymers obtained either by chemical synthesis or by chemical modification of natural polymers.

The poorly studied and unusual types of PHAs include PHA copolymers containing 4-hydroxyvalerate (4HV) monomers and polymers containing 3-hydroxy-4-methylvalerate (3H4MV) monomers [43]. These are three-component P(3HB-*co*-3HV-*co*-4HV) and P(3HB-*co*-3HV-*co*-3H4MV) polymers. There is fragmentary and sparse evidence that these types of PHA may be more technologically advanced than other types of PHA. Concerning P(3HB-*co*-3HV-*co*-3H4MV) copolymers, it has been shown that they are not subject to rapid “aging” and increased brittleness during storage [44,45,46], while P(3HB-*co*-3HV-*co*-4HV) copolymers have a reduced degree of crystallinity and glass transition temperature [47,48,49]. At the same time, information on the thermal behavior, thermal stability, and crystallization kinetics of these copolymers is extremely limited.

This determined the purpose of this work—a comparative study of the structural features and physicochemical properties of unusual P(3HB-*co*-3HV-*co*-4HV) and P(3HB-*co*-3HV-*co*-3H4MV) polymers, including the thermal stability, crystallization kinetics and spherulites formation, in comparison with the usual two-component 3-hydroxybutyrate and 3-hydroxyvalerate P(3HB-*co*-3HV) copolymers.

## 2. Results and Discussion

**Figure 1:**
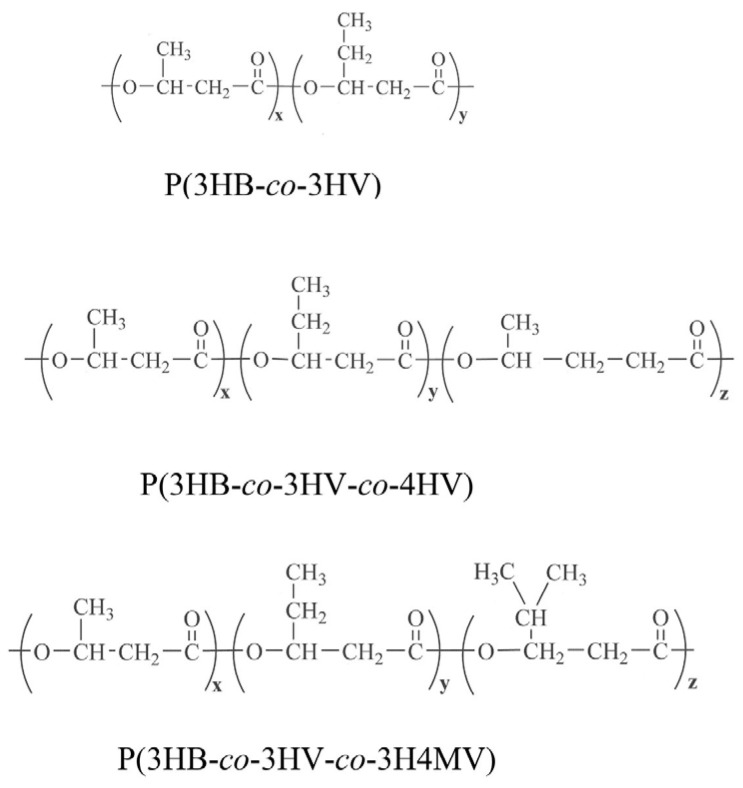
Structural formulas of PHA polymers studied.

A comparative study of the structural features and physicochemical properties of three types of PHA polymers containing in addition to 3HB monomers, 3HV, 4HV and 3H4MV at different ratios, was performed. The chemical compositions of the PHA polymers were identified using gas chromatography with mass spectra (Figure S1). Figure 1 shows the structural formulas for each type of PHA polymer.

The revealed differences in the structure of the three types of PHA were associated with differences in the chemical composition of the polymers studied, which differed in physicochemical properties. The properties of the polymers changed not only by the set of monomers but also by their ratio (Table 1).

Comparative studies of three types of copolymer PHA included studies of the degree of crystallinity, molecular weight and temperature characteristics. Particular attention was paid to the thermal behavior of copolymers of various chemical composition. For this purpose, the kinetics of crystallization and the formation of spherulites in three types of PHA polymers were studied for the first time in a comparative aspect.

### 2.1. Properties of P(3HB-co-3HV)

P(3HB-*co*-3HV) copolymers are isodimorphic due to the cocrystallization of monomer units [52]. Compared with P(3HB), these copolymers have lower crystallinity and melting point, lower rigidity, and higher elasticity [38,53,54,55,56,57].

GPC chromatograms of PHA samples illustrating molecular weight changes are given in Figure S2. The molecular weight indices (M_n_ and M_w_) for the P(3HB-*co*-3HV) samples varied in the ranges of 56–115 and 169–299 kDa, respectively, without a clear relationship with the content of 3HV monomers. The polydispersity index was similar (2.6–3.0). Similar results were obtained in [55,58,59]. The study of the crystallinity degree of P(3HB-*co*-3HV) showed a tendency for C_x_ to decrease from 66 to 56–58% as the content of 3HV monomers in the copolymer increased from 10.0 to 48.6 mol.%. This corresponded to the known data on the effect of 3HV monomers on the ratios of amorphous and crystalline phases in PHA [14,38,56]. The crystal structures of P(3HB-*co*-3HV) were very complex because the monomeric units of 3HB and 3HV could be partially incorporated into each other’s crystal lattices [60]. The ratios of the monomers determined the crystal structure of these copolymers and the process of crystallization into the 3-hydroxybutyrate or 3-hydroxyvalerate lattices due to isodimorphism [61,62]. The crystallinity and crystallization rate of P(3HB-*co*-3HV) changed with the 3HV monomers content increase [63,64,65,66].

**Figure 2:**
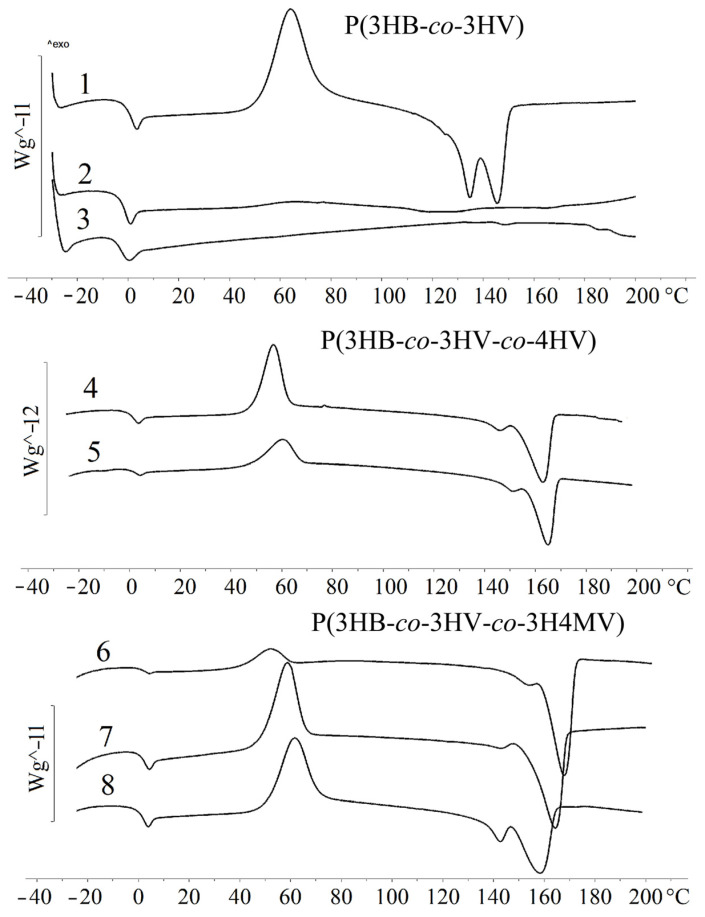
Temperature characteristics of the PHA samples (the numbering indicating that the composition of the copolymer is similar to the composition shown in Table 1).

The results of the study of the temperature characteristics of P(3HB-*co*-3HV) copolymers with different content of 3HV monomers are presented in Table 1 and in Figure 2.

The study of thermal characteristics (Table 1, Figure 2) showed that with an increase in the content of 3HV monomers, the melting point (T_melt_) decreased. The T_melt_ (145 °C) was recorded for a sample with a 3HV content of 10.0 mol.%. The sample with the highest content of 3HV monomers (48.6 mol.%) failed to crystallize under thermal analysis conditions. The melting point of 114 °C was obtained during the first heating, and characterizes the crystalline phase obtained by precipitation of the polymer from solution. The glass transition temperature (T_g_) decreased from −0.7 °C (10.0 mol.% of 3HV) to −4.6 °C (48.6 mol.% of 3HV). No correlation was found between the thermal degradation temperature (T_degr_) and the 3HV content in the copolymer. All samples showed good thermal stability. The gap between T_melt_ and T_degr_ was 139–152 °C. The obtained characteristics of the P(3HB-*co*-3HV) samples studied were generally comparable with the published results on the properties of P(3HB-*co*-3HV) [14,38,67].

**Figure 3:**
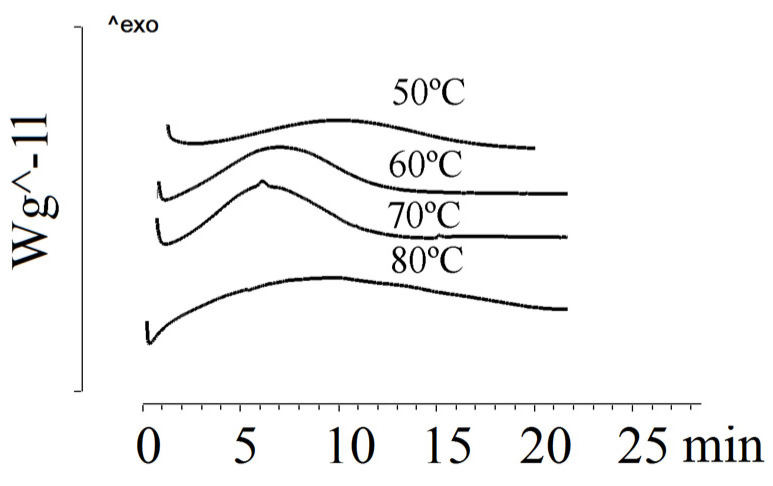
Crystallization of P(3HB-*co*-10 mol.%3HV) at different temperatures.

Figure 3 shows the crystallization of P(3HB-*co*-3HV) (3HV content of 10 mol.%) under isothermal conditions at various temperatures.

Under isothermal crystallization conditions, the P(3HB-*co*-3HV) sample with 10 mol.% of 3HV showed low crystallization rates. As the crystallization temperature increased, the crystallization time increased. If at 50 °C the crystallization time was 7.0 min, then at 90 °C it was 46.0 min. At 100 °C, the sample remained amorphous for 7 h. Blombergen et al. found that a melt-quenched P(3HB-*co*-3HV) sample containing 20 mol.% of 3HV remained amorphous after 15 min [62]. Under isothermal crystallization conditions, it was not possible to crystallize samples with a 3HV content of 26.2 mol.% and 48.6 mol.%. Difficulties in isothermal crystallization of mixtures with high 3HV content have been reported by Chan et al. [68].

### 2.2. Properties of P(3HB-co-3HV-co-4HV)

The molecular mass parameters of P(3HB-*co*-3HV-*co*-4HV) samples containing 4HV monomers of 2.3 and 4.8 mol.% and 3HV monomers of 9.4 and 22.9 mol.% differed insignificantly (Table 1). Mn values were 51 and 74 kDa; Mw values were 248 and 221 kDa. The polydispersity value was also similar (4.9 and 3.0). This was comparable with the data of [50], in which a series of P(3HB-*co*-3HV-*co*-4HV) samples with different ratios of 4HV, 3HV, and 3HB monomers had similar weight average molecular weights (201–248 kDa) with varying polydispersity from 2.9 to 4.9.

Comparison of the obtained results with published ones showed the ambiguity of the influence of the ratio of monomers in P(3HB-*co*-3HV-*co*-4HV) on the molecular weight. For example, in [47], samples with low 4HV inclusions (not higher than 1–2 mol.%) at different amounts of 3HB monomers (27 and 91 mol.%) showed higher values of Mw (510–570 kDa) compared with our work. In [48], even higher values of Mw (810 and 1060 kDa) were given for samples containing 2.5 and 6.7 mol.% of 3HV monomers and from 18.5 and 55.3 mol.% of 4HV monomers. Polymer samples in which the content of 3HV monomers was high (40.4 and 50.1 mol.%) and 4HV with no more than 4.8 mol.%, had super-high values of Mw (3624 and 4032 kDa) [49]. In general, published data do not allow unambiguous interpretation of the effect of the content of 3HV and 4HV monomers on the molecular weight of P(3HB-*co*-3HV-*co*-4HV).

The temperature characteristics of P(3HB-*co*-3HV-*co*-4HV (Table 1, Figure 2) were close to those of P(3HB-*co*-3HV). A feature of these copolymers was the presence of double peaks in the melting region. The polymer sample containing 3HB (9.4 mol.%) and 4HB (2.3 mol.%) monomers (sample 4 in Table 1) had the lowest T_melt_ (147 and 163 °C). Sample 5 with a high content of 3HB and 4HB monomers had a higher T_melt_, both peaks were recorded at 151 and 166 °C. The presence of two types of valerate monomers (3HV and 4HV) had the most significant effect on the T_g_, which decreased from 0.3 to −10.8 °C with an increase in their content. The sample with the lowest content of 3HV and 4HV monomers (9.4 and 2.3 mol.%) had a T_degr_ of 280 °C.

The information available in the literature was contradictory. For example, studies [47,49] showed no correlation between T_melt_ and T_degr_, but in [48] for samples with different contents of 3HV monomers, T_melt_ was significantly different. An increase in 4HV (18–47 mol.%) and 3HV (50–84 mol.%) monomers content led to a strong decrease in the T_melt_, but the T_g_ of the samples did not differ significantly [69].

The degree of crystallinity of the P(3HB-*co*-3HV-*co*-4HV) samples studied was 46 and 39% (Table 1), which was lower than that of P(3HB) and P(3HB-*co*-3HV). Earlier, when studying a series of these copolymers (six samples), amorphization was also shown when in addition to 3HB monomers, 4HV monomers were included in the composition of PHA, even in small amounts (from 1.9 to 4.7 mol.%). C_x_ values were below 50% and varied in the range of 38–49% [50].

**Figure 4:**
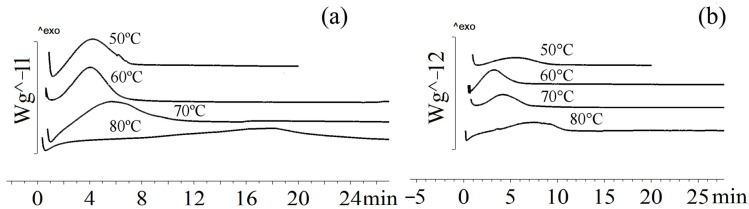
Crystallization of P(3HB-*co*-3HV-*co*-4HV) at different temperatures: (**a**) P(3HB-*co*-9.4 mol.%3HV-*co*-2.3 mol.%4HV), (**b**) P(3HB-*co*-22.9 mol.%3HV-*co*-4.8 mol.%4HV).

The crystallization rate of the sample containing 9.4 mol.% of 3HV and 2.3 mol.% of 4HV did not significantly differ from the crystallization rate of P(3HB-*co*-3HV) containing 10 mol.% of 3HV (Figure 4). At 50 °C, the crystallization of the sample occurred in 5.4 min., which was 30% faster than the crystallization of P(3HB-*co*-3HV) with a 3HV content of 10 mol.% at the same temperature. At temperatures of 60 °C and 70 °C, the crystallization of the sample occurred in 5–7 min, which was comparable to the crystallization rate of P(3HB-*co*-3HV) with a 3HV content of 10 mol.%. As the crystallization temperature increased to 80 and 90 °C, the crystallization time increased significantly to 13 and 42 min, respectively.

The crystallization rate increased with the increase in 3HV and 4HV monomers (sample 5 in Table 1). This sample was characterized by a decrease in the enthalpy of crystallization at temperatures of 50, 60, 70 °C to 36–37 J/g compared with a sample with a low content of valerate monomers (51–67 J/g), which may indicate a lower degree of crystallinity. There were no data in the literature on the isothermal crystallization of such copolymers.

### 2.3. Properties of P(3HB-co-3HV-co-3H4MV)

Unusual PHAs are methylated types of PHAs polymers that contain branched 3H2MV or 3H4MV monomers [39,40,41,44,45,70,71]. These works show the beneficial effects of these unusual monomers on the properties of PHA, including increased thermal stability and reduced crystallinity. Prof. M. Koller, in his extensive review work, emphasized that polymers containing 3H4MV monomers, in contrast with P(3HB) and P(3HB-*co*-3HV), have so far been little described; however, they are promising for various applications due to the fact that they are not subject to increasing crystallization and do not “age” over time, retaining their technological properties [43].

Previously, a series of P(3HB-*co*-3HV-*co*-3H4MV) polymers were synthesized by the C. necator B-10646 wild-type strain [51]. In the present work, samples with 3H4MV monomers content of 5.3 and 10.9 mol.% were obtained and studied at different contents of 3HV monomers, respectively, 23.4 and 8.6 mol.% (Table 1). The P(3HB-*co*-3HV-*co*-3H4MV) samples studied had higher weight average and number average molecular weights compared with P(3HB-*co*-3HV-*co*-4HV). With an increase in the content of 4HV monomers from 4.0 to 10.9 mol.%, the M_n_ values increased from 135 to 175 kDa, while the M_w_ value, on the contrary, was higher (769 kDa) in the sample with a lower content of 3H4MV monomers.

The study of the thermal properties of P(3HB-*co*-3HV-*co*-3H4MV) (samples 6–8 in the Table 1) showed a decrease in the T_melt_ with an increase in the content of 3H4MV monomers. The T_melt_ was recorded at 158 and 168 °C for a sample containing 8.0 mol.% of 3HV and 4.0 mol.% of 3H4MV (Table 1 and Figure 2). The minimum T_melt_ was recorded for a sample containing 23.4 mol.% of 3HV and 5.3 mol.% of 3H4MV (142 and 158 °C). The thermal behavior of methylated polymers was characterized by the presence of two peaks in the melting region. With an increase in 3HV and 3H4MV monomers, the T_g_ also decreased from 0.6 °C to −0.21 °C. During cooling, the crystallization peak was observed only in one sample with a minimum content of monomers in the region of 60 °C. Recrystallization of the sample was observed during subsequent heating to a temperature of 52 °C. The other samples, upon cooling, passed into the amorphous state and did not have crystallization peaks. In general, the samples, regardless of the content of 3H4MV and 3HV in them, showed the values of T_degr_ at the level of 295–297 °C. It was comparable to the values for P(3HB) homopolymer and P(3HB-*co*-3HV-*co*-4HV) polymers.

Published results showed that C_x_, T_melt_ and T_g_ values strongly decreased with increasing of 3H4MV monomers content from 0.35 to 0.47 mol.% [70]. Films obtained from these copolymers containing 3H4MV (1.3 and 18 mol.%) were distinguished by increased thermal stability and reduced crystallinity [72]. In [44], samples with a high (38 mol.%) content of 3H4MV monomers had M_n_ and polydispersity at a level from 90 × 10^3^ to 230 × 10^3^ Da and 1.3–1.8, respectively, and were medium strength lasting up to 180 days without significant deterioration. T_melt_ and the enthalpy of melting (ΔHm) decreased from 171 to 120 °C and from 74 to 13 J/g, respectively, with an 3H4MV content increase in the polymer. Ling et al. also obtained P(3HB-*co*-3HV-*co*-3H4MV) samples with a reduced degree of crystallinity [73].

**Figure 5:**
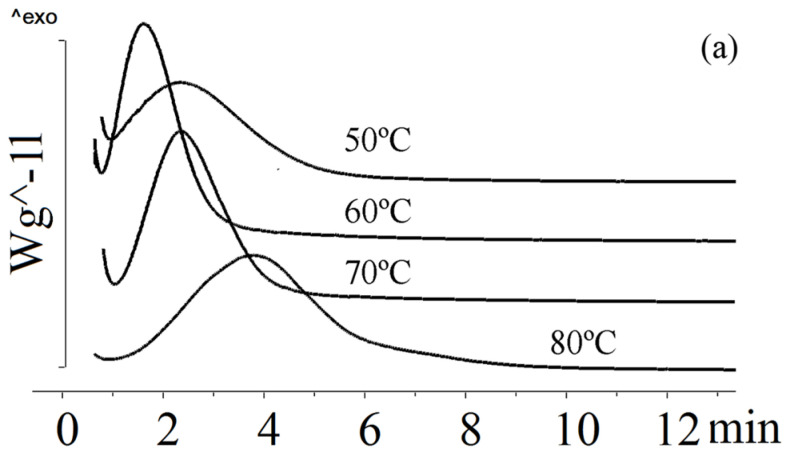

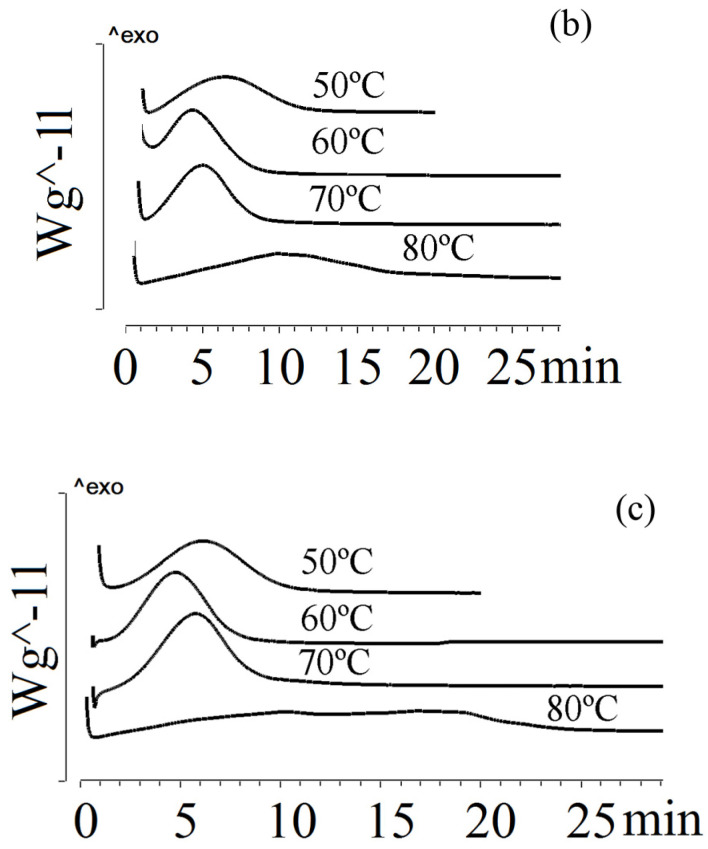
Crystallization of P(3HB-*co*-3HV-*co*-3H4MV) at different temperatures: (**a**) P(3HB-*co*-8.0 mol.%3HV-*co*-4.0 mol.%3H4MV), (**b**) P(3HB-*co*-8.6 mol.%3HV-*co*-10.9 mol.%3H4MV), (**c**) P(3HB-*co*-23.4 mol.%3HV-*co*-5.3 mol.%3H4MV).

The crystallinity of the P(3HB-*co*-3HV-*co*-3H4MV) copolymer samples was determined to be 40–49%. This was comparable to P(3HB-*co*-3HV-*co*-4HV) and exceeded that of P(3HB-*co*-3HV). Figure 5 illustrates the crystallization of P(3HB-*co*-3HV-*co*-3H4MV) at different temperatures.

All P(3HB-*co*-3HV-*co*-3H4MV) samples exhibited different behavior under isothermal crystallization conditions. The sample with the lowest total content of 3HV and 3H4MV monomers had the highest crystallization rate (sample 6 in Table 1). At 50 °C, its crystallization time was 3.6 min; at 60 °C, the rate increased and the crystallization time almost doubled. The longest crystallization time (9.9 min) was observed at 90 °C. This sample had the highest crystallization rate of all samples. The crystallization behavior of samples with significant differences in the content of 3HV and 3H4MV monomers (samples 7–8 in Table 1) was comparable. At 50 °C, the crystallization time was 7 min; at 60 and 70 °C it was 4.1 and 5.0 min, respectively. Subsequently, the crystallization rate decreased, and at 80 and 90 °C the crystallization time increased to 8.6 and 12.4 min. This was significantly less than that of P(3HB-*co*-3HV).

### 2.4. Formation of Spherulites during Isothermal Crystallization of PHA Polymers of Various Chemical Composition

**Figure 6:**
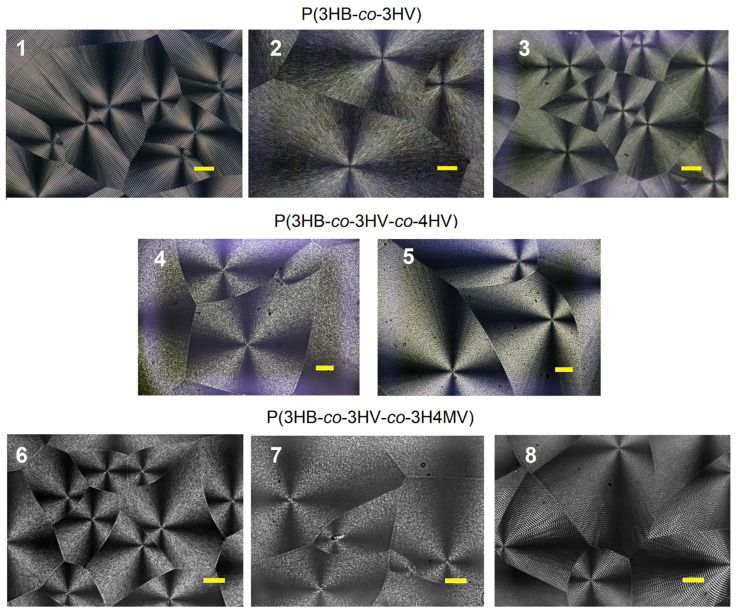
Examples of the morphology of spherulites formed during the exothermic crystallization of copolymer PHAs of various chemical compositions at 70 °C. Bar = 100 µm (numbering of sample compositions according to Table 1).

The most common crystalline form of polymers are spherulites, which are formed upon rapid cooling of melts or upon precipitation from a concentrated solution [74]. As a rule, semicrystalline PHAs are characterized by the formation of extremely large spherulites during crystallization [75], which occurs near the equilibrium state and in which crystalline lamellae develop into spherulites [76]. The isothermal crystallization kinetics of copolymer samples of various compositions was investigated by using differential scanning calorimetry (DSC) and hot-stage polarized optical microscopy (POM) in the temperature range of 60 °C to 100 °C. The morphology of spherulites formed during exothermal crystallization of copolymer PHAs of various chemical compositions is shown in Figure 6.

It was shown that the size of spherulites in P(3HB-*co*-3HV) samples depended on the temperature of isothermal crystallization and increased by a factor of 2–4 with an increase in temperature from 60 °C to 100 °C due to a decrease in the nucleation density. It is known that the T_melt_ of this copolymer varies depending on the content of 3HV monomers and the lattice type [77,78]. The morphology of the spherulites showed typical ring bands and a Maltese cross. At the same time, with an increase in the content of 3HV monomers, a decrease in the distance between the rings was noted.

The effect of twisting of lamellae in spherulites of a number of copolymers including P(3HB-*co*-3HV) was found earlier [79,80]. The authors concluded that the growth axis was one of the factors determining the twist handedness of the lamellae of chiral polymers. In the paper of Laycock and colleagues [81], the coarse and fine structures of spherulites obtained confirmed that P(3HB-*co*-3HV) is a mixture of nominally block and/or random copolymers with a very wide compositional distribution. It is known that the shape and size of spherulites in PHA are influenced by many factors, with the key factor being the temperature at which crystallization occurs, while the periodicity and regularity of the structure of spherulite bands can change depending on the conditions of crystallization, as well as the molecular weight of the polymer [82]. Ding and Liu [83] found that non-striped and double banded spherulites can form in PHA during isothermal crystallization from a thin melt at both low and higher crystallization temperatures.

The morphology of spherulites formed during isothermal crystallization of three-component P(3HB-*co*-3HV-*co*-4HV) also showed a standard Maltese cross (Figure 6). However, typical ring bands could not be found. With an increase in the crystallization temperature from 60 to 100 °C, the size of the spherulites increased. The maximum radius of spherulites reached 600–800 µm. The size distribution of spherulites was close for the two P(3HB-*co*-3HV-*co*-4HV) samples studied at all temperature ranges studied, despite the differences in the ratio of monomers (the content of 3HV and 4HV monomers between the samples differed by almost two times).

The morphology of spherulites formed during isothermal crystallization of methylated copolymers P(3HB-*co*-3HV-*co*-3H4MV) had differences (Figure 6). The Maltese cross was typical for all three studied samples with different ratios of monomers. However, the annular bands were clearly visible only in the sample with the lowest monomer content of 3HB and the highest content of 3HV (sample 8 in Table 1). The morphology of spherulites in PHA of this composition is presented in this work for the first time. The lack of such data in the available literature does not allow a comparison of the results.

**Figure 7:**
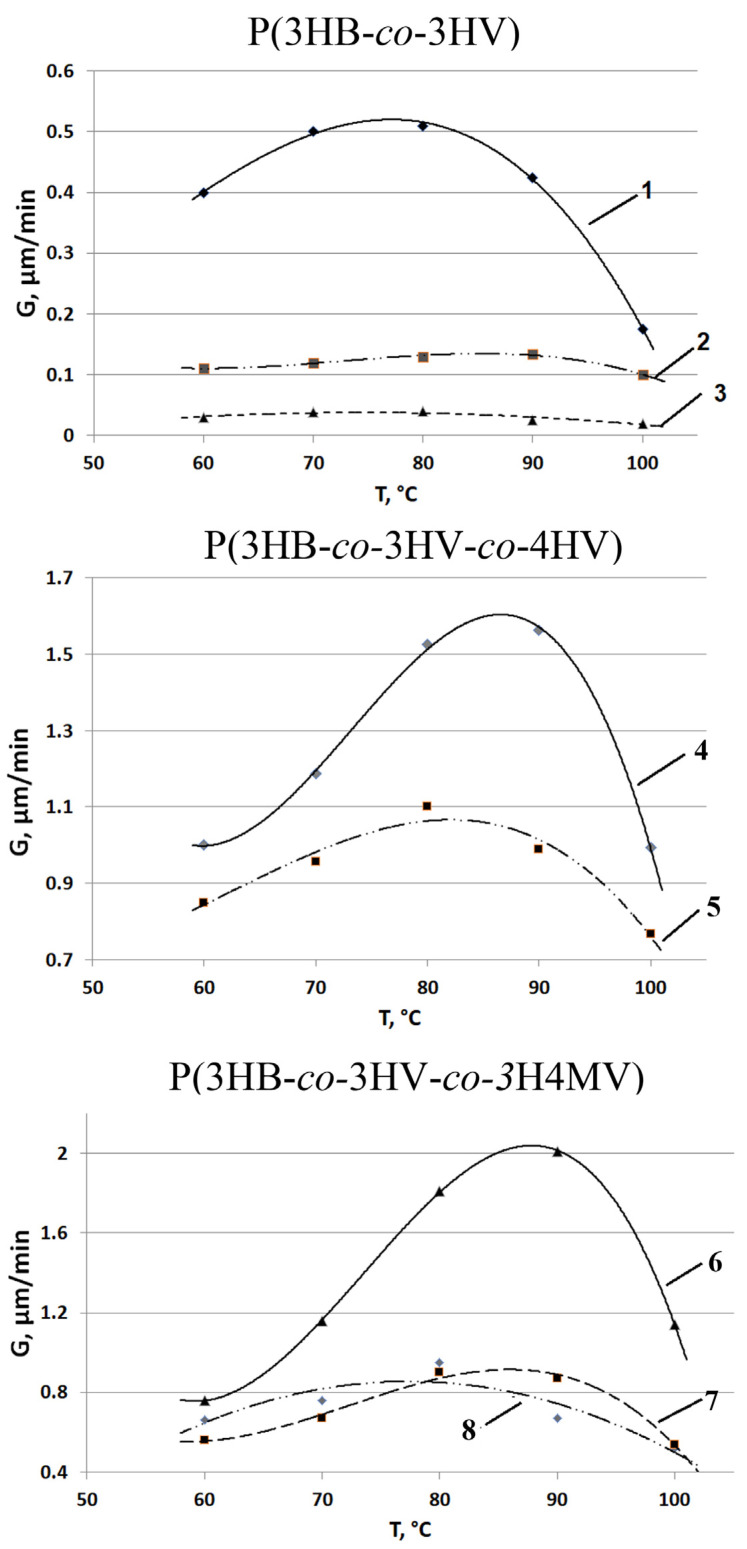
Dependence of the growth rate of spherulites (G) of PHA polymers of various chemical composition on the crystallization temperature (numbering of sample compositions according to Table 1).

It was found that the rate of formation of spherulites during isothermal crystallization of PHA samples depended on the type of copolymers and the ratio of monomers in them (Figure 7). The growth rate of spherulites in all types of copolymers significantly depended on the ratio of monomers. Thus, the maximum growth rate of spherulites (Gmax) among the investigated P(3HB-*co*-3HV) was the highest (0.52 µm/min) for the sample with the lowest content of 3HV monomers (10.0 mol.%), i.e., the one closest to the P(3HB) homopolymer. This significantly exceeded the index for samples with a higher content of 3HV (26.2 and 48.6 mol.%), 0.3 and 0.12 µm/min, respectively, at the same temperature of the isothermal crystallization process.

Consequently, the growth rate of spherulites decreased with an increase in the content of 3HV monomers in P(3HB-*co*-3HV) copolymers. This corresponded to the results described earlier [84], where it was shown that the value of the maximum growth rate of spherulites during isothermal crystallization has an inverse dependence on the content of 3HV monomers. A similar effect was recorded in a series of studies that showed that in P(3HB-*co*-3HV), as compared with P(3HB) homopolymers, the crystallization rate curve shifted to the low-temperature region, while the nucleation rate dropped sharply [54,62,85]. Laycock and colleagues [81] showed that with an increase in the content of 3HB monomers, crystallization increased more actively at a lower temperature compared with samples with a low content of 3HV, in which the maximum crystallization rates were achieved in the range between 70 and 90 °C. Another study [86] showed that the growth rate of spherulites in P(3HB-*co*-3HV) had a maximum value (about 353 K) and its crystallization ability was lower than that of P(3HB). In a review [87], it was shown that, according to the data of [86], the maximum rate of spherulite formation of 1.0 µm/min was typical for P(3HB-*co*-3HV) copolymers with a content of 3HV monomers of 8.0 mol.% at 70–80 °C. This was close to P(3HB) [88], but was orders of magnitude inferior to the values for short-chain P(3HB-*co*-4HB) [89] and medium-chain P(3HB-*co*-3HHx) polymers [90].

Higher spherulite growth rates were recorded for two samples of P(3HB-*co*-3HV-*co*-4HV) polymers (Figure 7). In this case, the highest value (1.6 µm/min) was characteristic of the sample 5 in Table 1, in which the content of 3HV and 4HV monomers was two times higher than the content of these monomers in another sample, and its G value was at the level of 1.1 µm/min. The dependence of the maximum growth rate of spherulites for these copolymers containing, in addition to 3HB monomers, two types of valerate monomers, 3HV and 4HV, showed a shift to the high temperature (positive) side with an increase in the content of 3HV and 4HV. Thus, Gmax for P(3HB-*co*-3HV-*co*-4HV) exceeded that of P(3HB-*co*-3HV) by one to two orders of magnitude, depending on the ratio of monomers.

The temperature dependence of the maximum growth rate of spherulites G(T) for the methylated P(3HB-*co*-3HV-*co*-3H4MV) samples studied was bell-shaped with a maximum value in the temperature range of 80–90 °C. The value was highest (2.1 µm/min) for the sample with the highest 3HB monomer content and the lowest 3HV monomer content (sample 6 in Table 1). Two other samples (7 and 8 in Table 1) with a significant difference in the content of the 3H4MV (5.3 and 10.9 mol.%) and 3HV (23.4 and 8.6 mol.%) monomers, regardless of this, had a similar shape of the dependence G(T) and close quantitative indicators, namely, the maximum growth rate of spherulites (0.6 and 0.8 µm/min). A common characteristic of the dependences G(T) for this type of copolymers is the shift of the extremum (maximum velocity) to the high temperature side. This probably indicates the predominant influence on the growth rate of spherulites of the chemical composition of the samples.

As is known, the growth rate of spherulites depends on the primary nucleation. Primary nucleation is often a rather complex process, since it depends on the crystallization temperature, the density of inhomogeneities, and the presence/absence of nucleating agents [14]. In this regard, it should be noted that during the crystallization process for both P(3HB-*co*-3HV-*co*-4HV) and P(3HB-*co*-3HV-*co*-3H4MV) polymers, the number of spherulite nuclei increased insignificantly. This apparently indicates the thermal character of nucleation. The heterogeneity of the sizes of spherulites was fixed at the end of the process of isothermal crystallization of both types of copolymers, regardless of their composition.

Thus, the polymers studied, differing in the set and ratio of monomers, including three types of different valerate monomers, had some differences in the morphology of spherulites and in the rate of their formation. The highest G values were recorded for three-component polymers of both types (from 0.6–0.8 to 1.6–2.1 µm/min). Morphological differences between three-component polymers require further research.

### 2.5. Structure and Surface Properties of Films Obtained from PHAs Polymers of Various Chemical Composition

The chemical composition, along with processing technologies, has a great influence on the properties of polymer products. At the same time, porosity, roughness, and hydrophilicity affect the adhesion properties of the surface, determining attachment, subsequent cell differentiation, and the course of reconstructive tissue genesis [91]. The effect of the chemical composition of PHA on the functional properties of biomedical products has been studied in a number of works [92,93,94,95]. However, data on the properties of films made from P(3HB-*co*-3HV-*co*-4HV) and P(3HB-*co*-3HV-*co*-3H4MV) polymers are extremely limited. In this work, for the first time, a comparative study of the properties of the surface of films obtained from these types of PHA was carried out.

**Figure 8:**
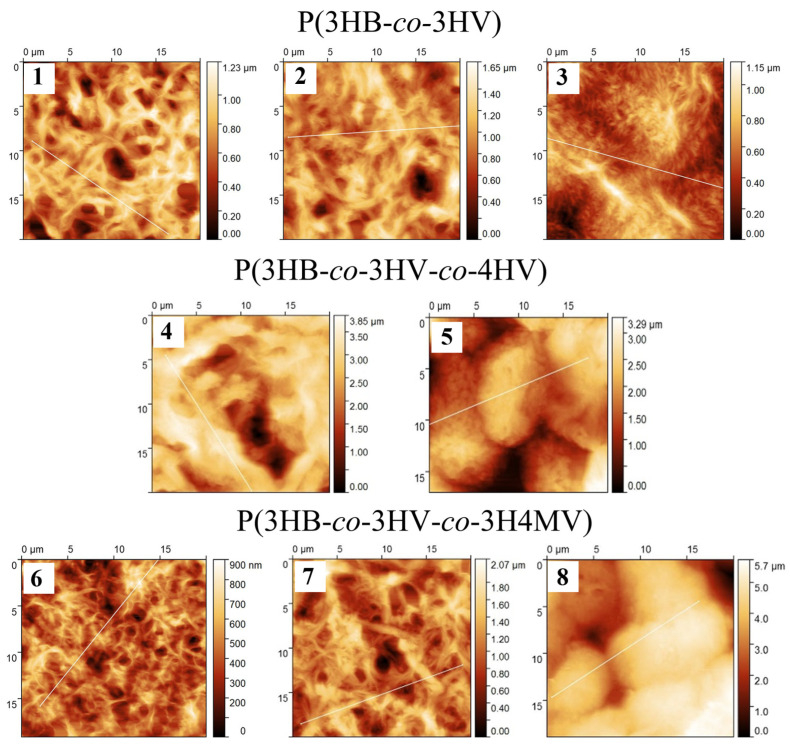
AFM images of naive films made from PHAs of various compositions (numbering of the sample compositions is according to Table 1; Table 2).

The results of the study of the roughness of film samples according to the results of atomic force microscopy are presented in Table 2 and Figure 8. The results of studying the roughness of films based on the results of atomic force microscopy are presented in Table 2 and Figure 8. The arithmetic mean (Sa) and root mean square (Sq) roughness were found. The integral indicator was the maximum height (Sz), including the entire range of values between the minimum and maximum of profile irregularities (between the maximum trough depth (Sv) and maximum peak height (Sp)).

The results obtained (Table 2) showed the difference in quantitative indicators of roughness between the three studied groups of copolymer PHA, but did not reveal a relationship between the ratio of monomers and roughness indicators within the studied groups. It was shown that all three samples of P(3HB-*co*-3HV) copolymers had the lowest values of arithmetic mean (Sa) and root mean square roughness (Sq), in the range of 141–172 and 178–205 nm, respectively, as well as in terms of the integral index (maximum height (Sz), 1123–1447 nm). Differences in the surface morphology of the films were revealed within this group of copolymers at different contents of 3HV monomers formed by wide and long “fibers”. With an increase in 3HV monomers to 48.6 mol.%, the surface was formed by narrow “fibers” without obvious pits or depressions, however, clearly pronounced “ridges” were observed at the tops of large fibers.

Both groups of three-component polymers had higher values of all studied roughness indices. The exception was one sample of the methylated copolymer P(3HB-*co*-3HV-co-3H4MV), in which the content of the 3HV and 3H4MV monomers was the lowest of the studied three samples of this group, 8.0 and 4.0 mol.%, respectively. The values of Sq, Sa, and Sz for this sample were at the level of those of P(3HB-*co*-3HV) copolymers. The highest roughness values were for two other samples from the P(3HB-*co*-3HV-*co*-3H4MV) group, for Sq and Sa, respectively, 332–933 and 253–822 nm, as well as for the maximum height index (Sz), which was determined at 2370.0 and 5348.5 nm. For films made of methylated copolymers, the surface was formed by “tangled” fibers with inclusions. On the surface there were large depressions with sharp (vertical) edges. The surface of the sample with the highest content of 3HV monomers (23.4 mol.%) was formed by rounded formations. The sample that was noted above with roughness indices comparable to those of P(3HB-*co*-3HV) was the smoothest and had the largest developed surface. The average position in terms of roughness between methylated copolymers and P(3HB-*co*-3HV) was occupied by three-component P(3HB-*co*-3HV-*co*-4HV) samples. The surface of the films from these polymers had extremely deep depressions–pores, the depth of which was not reliably determined and the statistical values were determined only using a mask (75% of the total height).

The roughness indices of a series of PHAs of various compositions studied earlier [96] showed differences. The roughness of the P(3HB) homopolymer was at the level of 144, 181, and 1241 nm for Sa, Sq, and Sz, respectively, according to the parameters studied. This was several times lower than that of the P(3HB-*co*-3HV) polymers, in which the content of 3HV monomers in the P(3HB) chain increased the surface roughness of the films.

The hydrophilic/hydrophobic balance of the surface is a significant parameter that indirectly characterizes the hydrophilicity of the sample, influencing the adhesive properties of the surface. An indicator of this ratio is the value of the contact angle of wetting the surface with liquids. Table 3 shows the surface characteristics of films obtained from PHA polymers of various chemical composition.

A clear relationship between the inclusion of certain monomers in the PHA polymers and the value of the contact angles was not established due to a significant scatter of values. Somewhat higher values (up to 95–104°) of the water smearing angle for films made from P(3HB-*co*-3HV) and P(3HB-*co*-3HV-*co*-4HV), and slightly lower values (80–90°) for films obtained from methylated P(3HB-*co*-3HV-*co*-3H4MV) polymers, were noted. For three-component polymers, a tendency to some decrease in free surface energy and dispersion component was noted. On the contrary, when diiodomethane (CH_2_I_2_) was used as a wetting liquid, the values of the contact angle were lower for two-component P(3HB-*co*-3HV) (44–49°) compared with the index for three-component polymers (56–60°). The values of the polar component were the highest for methylated polymers (2.8–5.5 mN/m). This significantly outperformed values for (3HB-*co*-3HV) and P(3HB-*co*-3HV-*co*-4HV) polymers.

In general, the results showed that in the presence of 3HV, 4HV, and 3H4MV monomers in the 3-hydroxybutyrate chain, there was a tendency to decrease the contact angle of wetting with water and an increase in the roughness parameters.

## 3. Materials and Methods

### 3.1. PHA Producer Strain and Cultivation Media

Polymers were synthesized using the *Cupriavidus necator* B-10646 wild-type strain registered in the Russian National Collection of Industrial Microorganisms (VKPM) [97]. The strain is capable of synthesizing PHA from various carbon substrates. The culture was grown in the mineral Schlegel medium [98]—a strong phosphate-buffered solution. The main carbon source was fructose (Panreac, Barcelona, Spain). To synthesize PHA copolymers, the cell culture was supplemented with precursors of sodium valerate (Sigma-Aldrich, Saint Louis, MO, USA), γ-valerolactone (Sigma-Aldrich, Saint Louis, MO, USA) or sodium 4-methylvalerate (Sigma-Aldrich, Saint Louis, MO, USA).

### 3.2. PHA Analysis

The copolymers were extracted from the cell biomass with dichloromethane (Ekos-1, Staraya Kupavna, Russia); the resulting extract was concentrated on an R/210V rotary evaporator (Büchi, Flawil, Switzerland) and then precipitated with ethanol (Ekos-1, Staraya Kupavna, Russia). Repeating the procedures of polymer dissolution and reprecipitation ensured the removal of impurities and obtaining homogeneous samples. The copolymer samples were dried in a fume hood at room temperature for 72 h.

The composition of each polymer was determined by chromatography of methyl esters of fatty acids after methanolysis of purified polymer samples using a 7890A chromatograph-mass spectrometer (Agilent Technologies, Santa Clara, CA, USA) equipped with a 5975C mass detector (Agilent Technologies, Santa Clara, CA, USA).

### 3.3. PHA Properties

The physicochemical properties of the polymers were examined using high-performance liquid chromatography and differential scanning calorimetry. All methods have previously been described in detail [99].

Molecular weight and molecular weight distribution of PHA polymers were examined using a gel permeation chromatograph (Agilent Technologies 1260 Infinity, Waldbronn, Germany) with a refractive index detector, using an Agilent PLgel 5 μm Mixed-C 300 × 7.5 mm column (Agilent Technologies, Church Stretton, UK). Chloroform (Ekos-1, Staraya Kupavna, Russia) was the eluent, at a flow rate of 1.0 mL/min at 40 °C. Typical sample volumes were 50 µL at a polymer concentration of 5 mg/mL. Polystyrene standards (Agilent Technologies, Church Stretton, UK) were used for the calibration procedure. Weight average molecular weight (M_w_), number average molecular weight (M_n_) and polydispersity (Ð = M_w_/M_n_) were determined.

Thermal analysis was performed using a DSC-1 instrument (Mettler Tolledo, Schwerzenbac, Switzerland). A sample of 3–4 mg was heated to 200 °C at a rate of 20 °C/min, then cooled to −30 °C at a rate of 20 °C/min, and heated again to 200 °C. The glass transition, melting point and pre-crystallization temperatures were taken from the second heating.

Isothermal crystallization was carried out on a DSC-1 instrument (Mettler Tolledo, Schwerzenbac, Switzerland). A sample of 3–4 mg was heated to 200 °C at a rate of 20 °C/min, and quenched at 200 °C for 2 min, then cooled at a rate of 50 °C/min to the specified crystallization temperature (50, 60, 70, 80, 90 °C). After crystallization, the sample was heated to 200 °C at a rate of 20 °C/min.

X-ray diffraction analysis using a D8 ADVANCE X-ray powder diffractometer and a VANTEC fast linear detector (Bruker AXS, Karlsruhe, Germany) was carried out to determine the crystallinity of PHA. Crystallinity (C_x_) was detected as the ratio of the area under the radiograph with the subtracted amorphous background to the area without subtracting the background. The Eva program from the diffractometer software application was applied for calculations.

### 3.4. Optical Studies of the Spherulites Formation

The morphology and radial growth rate of the spherulites of the PHA samples subjected to isothermal crystallization at different temperatures were observed using a polarizing optical microscope (POM) (Nikon, Eclipse E600 POL, Tokyo, Japan) equipped with a heating stage (hot stage) (Linkam LTS420, Redhill, UK). The samples were first melted at 195 °C for 3 min to destroy the thermal prehistory, and then cooled to the desired (studied) crystallization temperature. The growth rate of the spherulites (G) was calculated by measuring the radius, R, as a function of crystallization time, t, and expressed in μm/min. The observation continued until the field of view was completely covered by spherulites.

### 3.5. Production and Investigation of Polymer Films

Films of PHA polymers were prepared by casting chloroform solutions (1–2%) heated to 35 °C on degreased glass, and subsequent drying for 48 h in a dust-free box (Labconco, Kansas, MO, USA).

Contact angles of wetting with water of films were studied using a DSA-25E drop shape analyzer (Krüss, Heidelberg, Germany) and the DSA-4 software for Windows.

The roughness of film surface was determined using atomic force microscopy (AFM) in semicontact mode (DPN 5000, NanoInk, Skokie, IL, USA). Scanning areas 20 × 20 μm were carried out for 3–4 different areas of the surface for each of the samples. AFM data were processed and statistical analysis of the images was performed using the Gwyddion (2.51) free software. The following statistical values were determined: root mean square roughness (Sq), arithmetic mean surface roughness (Sa), maximum height (Sz), and Rq, the root mean square roughness value taken from the section profile.

## 4. Conclusions

The paper presents the results of a study of the physicochemical properties, features of thermal behavior and crystallization of polymers containing various types of valerate monomers depending on the set and ratio of monomers. The properties of the synthesized three-component copolymers of two types, P(3HB-*co*-3HV-*co*-4HV) and P(3HB-*co*-3HV-*co*-3H4MV) containing unusual monomers 4-hydroxyvalerate (4HV) and 3-hydroxy-4-methylvalerate (3H4MV), in addition to the usual monomers 3-hydroxybutyrate (3HB) and 3-hydroxyvalerate (3HV), were studied. It was shown that both types of terpolymers tend to increase thermal stability, especially for methylated samples, including an increase in the gap between T_melt_ and T_degr_; and an increase in the melting point and glass transition temperatures, as well as a lower degree of crystallinity (C_x_ 40–46%) compared with P(3HB-*co*-3HV) (58–66%). Measurement of the kinetics of crystallization of polymer samples in a wide temperature range showed that the kinetics of crystallization depends on the chemical composition of the samples, and the set and ratio of monomers in them. The features of exothermic crystallization of three- and two-component polymers were studied. It was shown that in terpolymers during exothermic crystallization, the formation of spherulites occurs at a high rate. The G_max_ value, depending on the set and ratio of monomers 3HV, 4HV, and 3H4MV, reached 1.6–2.0 µm/min. This was several times higher than the G_max_ index (0.52 µm/min) for the P(3HB-*co*-3HV) copolymer. The revealed differences in the thermal properties and crystallization kinetics of terpolymers are important for improving the processing technologies of polymers of the degradable polyhydroxyalkanoates family and indicate the promise of P(3HB-*co*-3HV-*co*-4HV) and P(3HB-*co*-3HV-*co*-3H4MV) for processing into quality products from melts, e.g., for degradable packaging, greenhouse and mulching films. The unusual three-component polymers studied can also be considered as a promising material for drugs delivery and the production of functional low-crystalline scaffolds for cell and tissue engineering technologies, etc.

## Figures and Tables

**Table 1:** Composition and physicochemical properties of PHA with different sets and ratios of 3HB, 3HV, 4HV and 3H4MV monomers synthesized by the wild-type *Cupriavidus necator* B-10646 strain.

N	Composition of Monomers, mol.%			M_n_, kDa	M_w_, kDa	Ð	C_x,_ %	T_melt_, °C	T_degr_, °C	T_g_, °C	T_cryst_, °C
	3HB	3HV									
1	90.0	10.0		89	233	2.6	66	134 145	284	−0.7	64
2	73.8	26.2		56	169	3.0	56	125	277	−2.5	63
3	51.4	48.6		115	299	2.6	58	114 *	283	−4.6	-
	3HB	3HV	4HV								
4 **	88.3	9.4	2.3	51	248	4.9	46	147 163	280	0.3	71.2 55.9
5	72.3	22.9	4.8	74	221	3.0	39	151 166	264	−10.8 1.51 0.69	55.2 61.9
	3HB	3HV	3H4MV								
6 ***	88.0	8.0	4.0	135	769	5.7	43	158 168	295	0.51	60 52
7	80.5	8.6	10.9	175	676	3.9	40	142 164	296	0.22	59.0
8	71.3	23.4	5.3	91	197	2.2	49	142 158	297	−0.21	61.5

* data from the first heating, ** the sample was synthesized earlier [50], *** the sample was synthesized earlier [51].

**Table 2:** Roughness indices of various types of PHA copolymers according to atomic force microscopy (AFM) data.

N	Composition of Monomers, mol.%			Sq, nm	Sa, nm	Rq, nm	Sz, nm
	3HB	3HV					
1	90.0	10.0		205.3	165.2	134.3	1296.5
2	73.8	26.2		216.5	172.1	157.2	1447.3
3	51.4	48.6		178.3	141.5	100.6	1123.5
	3HB	3HV	4HV				
4	88.3	9.4	2.3	571.1	455.2	472.4	3171.0
5	72.3	22.9	4.8	401.1	324.7	298.7	2208.7
	3HB	3HV	3H4MV				
6	88.0	8.0	4.0	129.3	102.9	122.2	940.5
7	80.5	8.6	10.9	332.9	253.1	324.3	2370.0
8	71.3	23.4	5.3	993.0	822.0	648.2	5348.5

**Table 3:** Surface properties of cast films obtained from PHA polymers of various chemical composition.

N	Composition of Monomers, mol.%			Contact Angle of Wetting H_2_O, °	Contact Angle of Wetting CH_2_I_2_, °	Free Surface Energy, mN/m	Dispersion Component, mN/m	Polar Component, mN/m
	3HB	3HV						
1	90.0	10.0		89.9 ± 0.6	44.9 ± 0.6	38.6 ± 0.2	37.1 ± 0.1	1.5 ± 0.0
2	73.8	26.2		104.6 ± 1.4	49.4 ± 0.8	34.6 ± 0.2	34.6 ± 0.2	0.0 ± 0.0
3	51.4	48.6		95.0 ± 1.3	43.8 ± 0.9	38.2 ± 0.2	37.6 ± 0.2	0.6 ± 0.0
	3HB	3HV	4HV					
4	88.3	9.4	2.3	94.8 ± 1.0	59.6 ± 1.4	30.4 ± 0.3	28.8 ± 0.3	1.6 ± 0.1
5	72.3	22.9	4.8	104.0 ± 1.2	59.2 ± 1.1	29.2 ± 0.2	29.0 ± 0.2	0.2 ± 0.0
	3HB	3HV	3H4MV					
6	88.0	8.0	4.0	80.9 ± 1.4	56.0 ± 1.4	36.4 ± 0.4	30.9 ± 0.3	5.5 ± 0.1
7	80.5	8.6	10.9	85.4 ± 1.5	60.0 ± 0.9	32.9 ± 0.3	28.6 ± 0.2	4.4 ± 0.1
8	71.3	23.4	5.3	90.0 ± 1.2	59.2 ± 1.0	31.8 ± 0.2	29.0 ± 0.2	2.8 ± 0.1

## Data Availability

All data are available in the paper.

## Supplementary Materials

The following supporting information can be downloaded at: https://www.mdpi.com/article/10.3390/ijms241814082/s1.
