# Supplementary figures and images for: Biodegradable Polyhydroxyalkanoates with a Different Set of Valerate Monomers: Chemical Structure and Physicochemical Properties
Source: Int J Mol Sci. 2023 Sep 14;24(18):14082. doi: 10.3390/ijms241814082 (PMC10531092; doi:10.3390/ijms241814082)

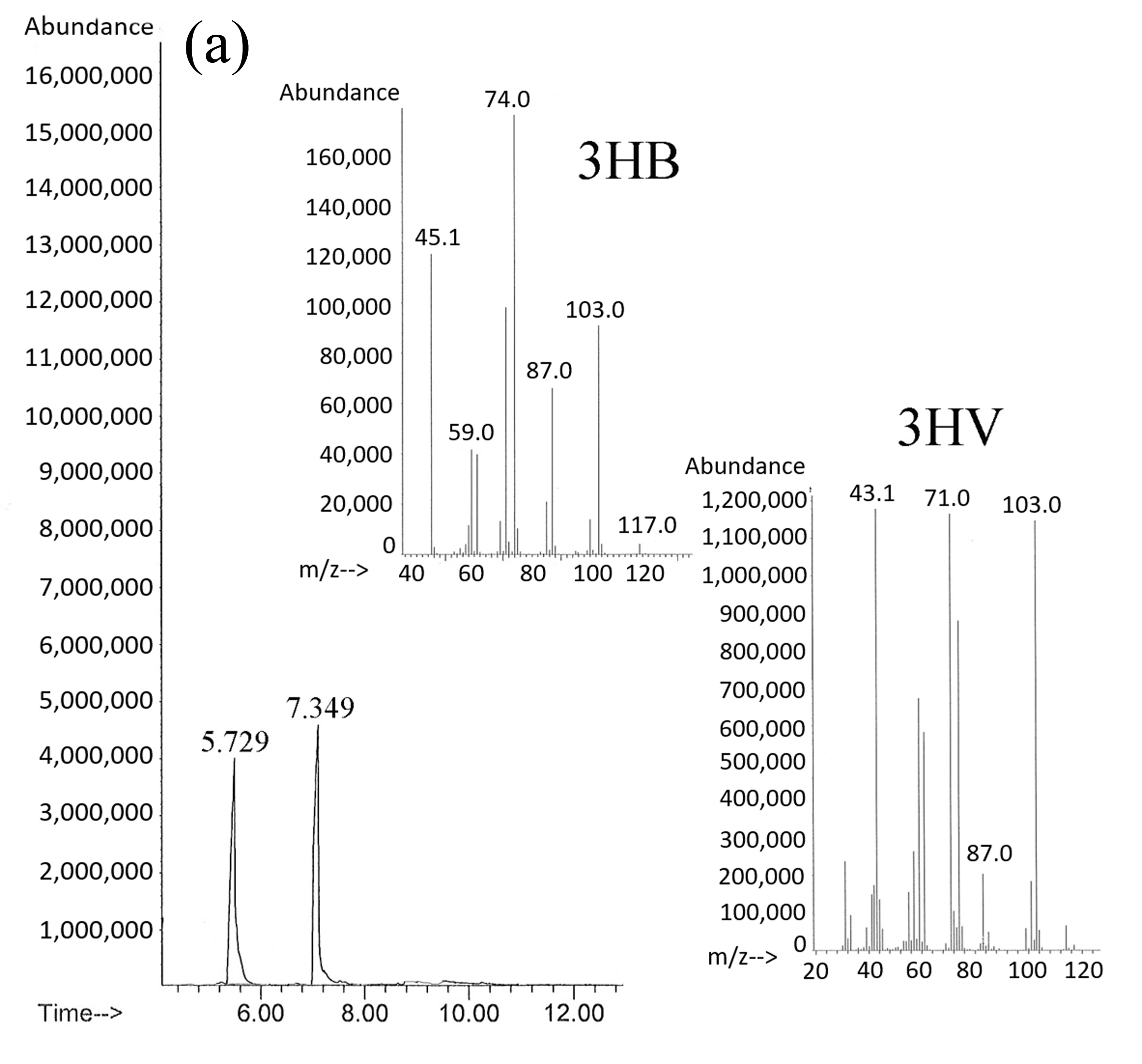

Supplement: Supplementary file 1 [file ijms-24-14082-s001.zip › Figure S1a.tif]

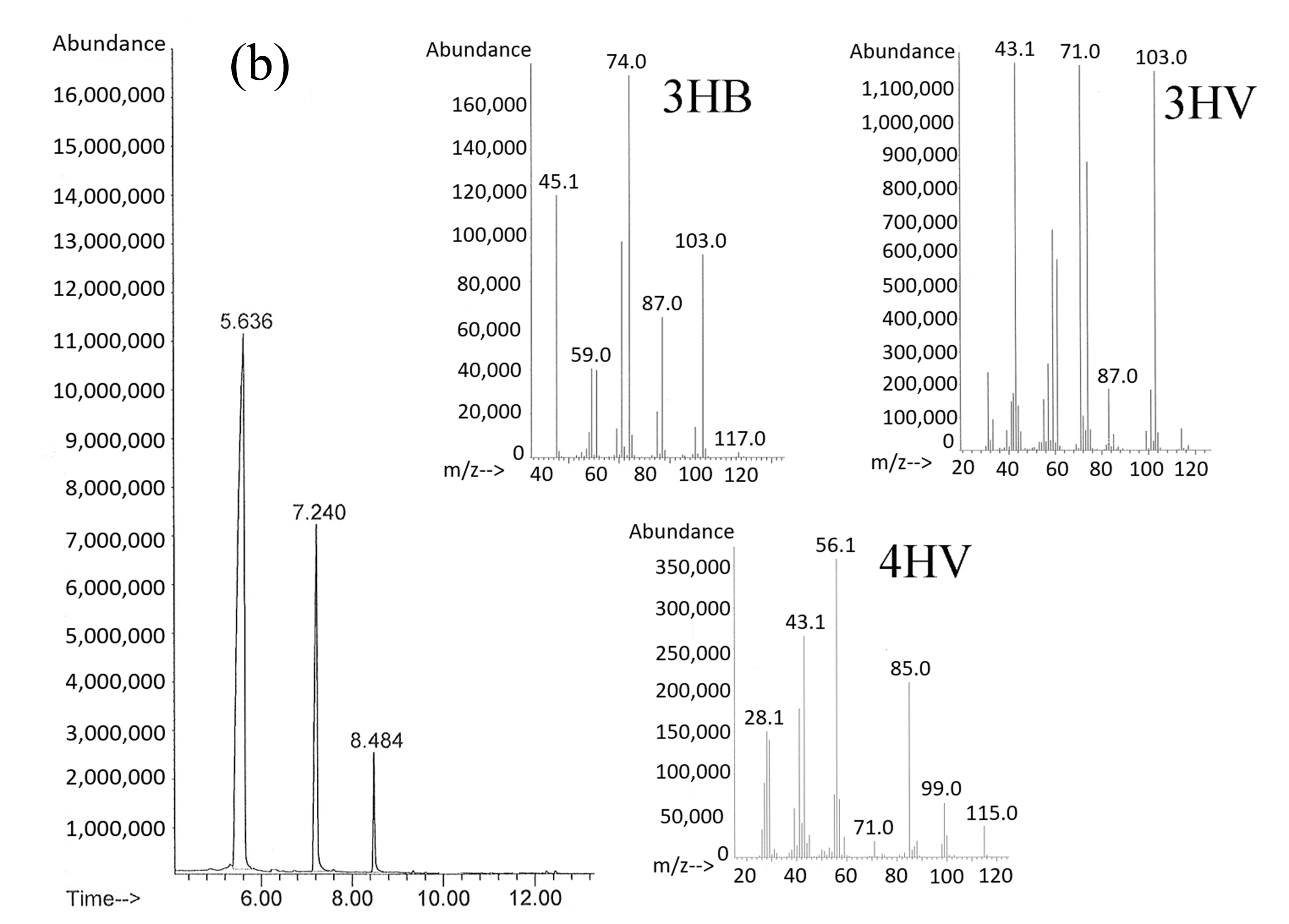

Supplement: Supplementary file 1 [file ijms-24-14082-s001.zip › Figure S1b.tif]

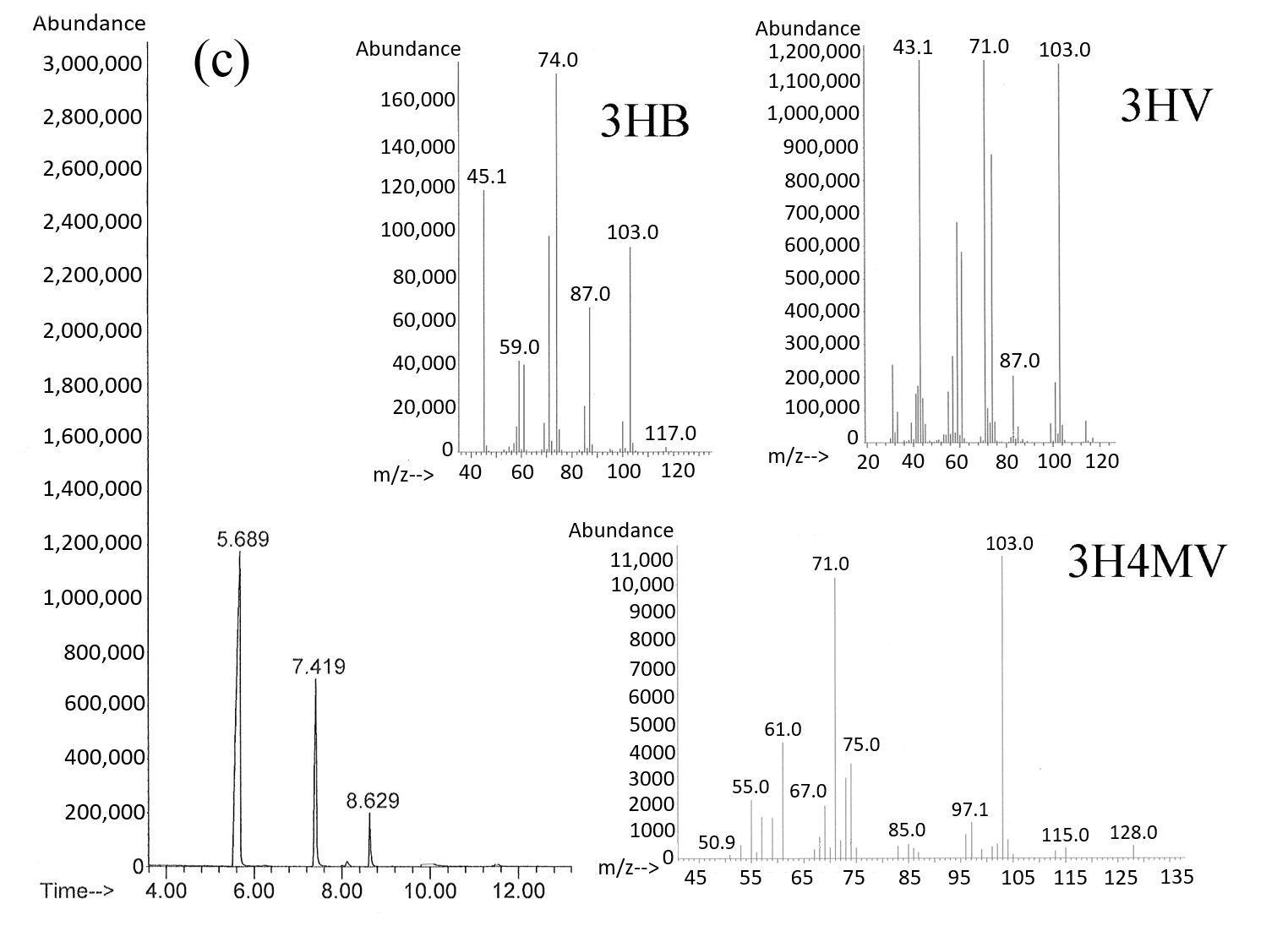

Supplement: Supplementary file 1 [file ijms-24-14082-s001.zip › Figure S1c.tif]

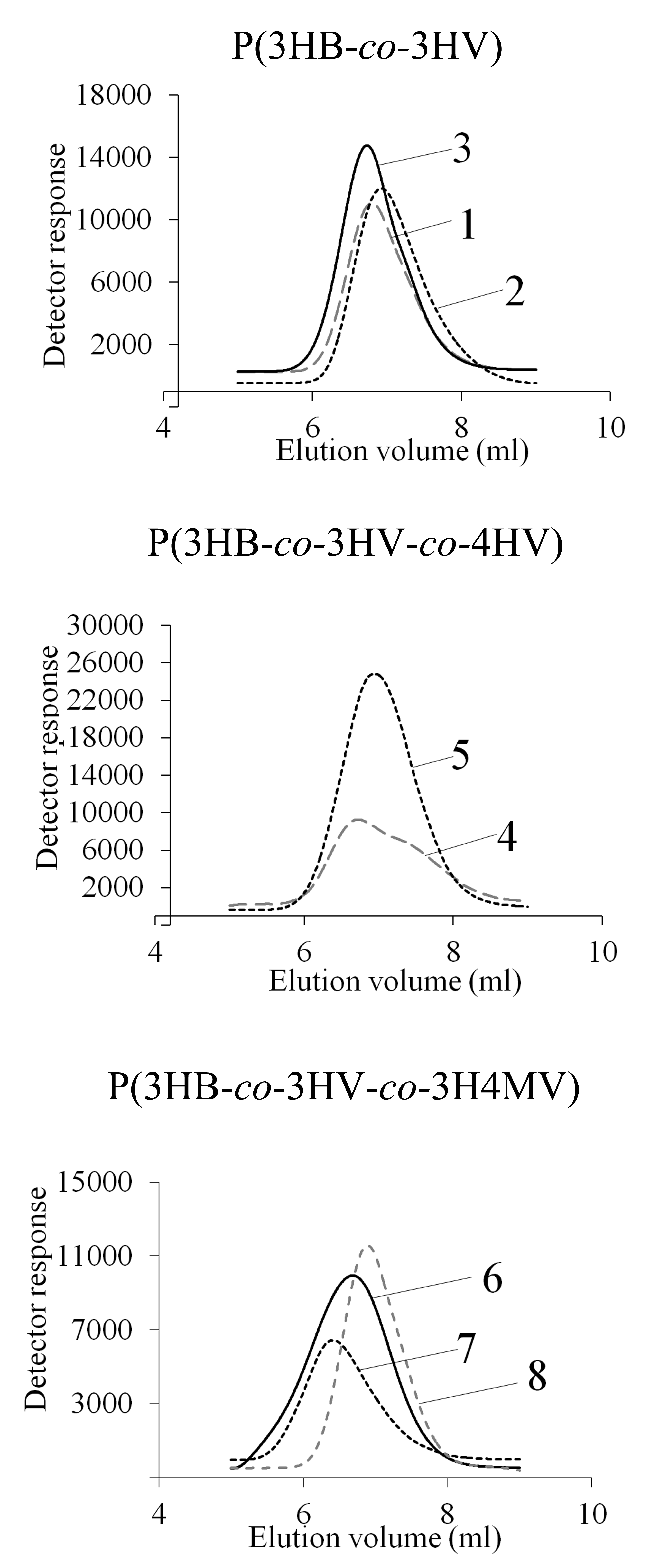

Supplement: Supplementary file 1 [file ijms-24-14082-s001.zip › Figure S2.tif]
